# Failure to shorten the diagnostic delay in two ultra-orphan diseases (mucopolysaccharidosis types I and III): potential causes and implications

**DOI:** 10.1186/s13023-017-0733-y

**Published:** 2018-01-08

**Authors:** Gé-Ann Kuiper, Olga L. M. Meijer, Eveline J. Langereis, Frits A. Wijburg

**Affiliations:** Department of Pediatric Metabolic Diseases, Emma Children’s Hospital and Amsterdam Lysosome Center “Sphinx”, Academic Medical Center, Meibergdreef 9, 1105 AZ Amsterdam, The Netherlands

**Keywords:** Mucopolysaccharidosis type I, Mucopolysaccharidosis type III, Diagnostic delay, Awareness, Rare diseases

## Abstract

**Background:**

Rare diseases are often un- or misdiagnosed for extended periods, resulting in a long diagnostic delay that may significantly add to the burden of the disease. An early diagnosis is particularly essential if a disease-modifying treatment is available. The purpose of this study was to assess the extent of the diagnostic delay in the two ultra-rare diseases, i.e., mucopolysaccharidosis I (MPS I) and III (MPS III), both of which are lysosomal storage disorders with different phenotypic severities (MPS 1 is characterized by the severe Hurler and the more attenuated non-Hurler phenotypes, MPS III is characterized by the severe rapidly progressing (RP) phenotype and more attenuated slowly progressing (SP) phenotype). We investigated whether the diagnostic delay changed over the previous decades.

**Results:**

The diagnostic delay, which is defined as the time between the first visit to a medical doctor for disease-related symptoms and the final diagnosis, was assessed using telephone interviews with patients diagnosed between 1988 and 2017 and/or their parents or legal guardian(s). In addition, the medical charts were reviewed. For MPS I (*n* = 29), the median diagnostic delay was 8 months (range 1-24 months) for Hurler patients and 28 months (range 2-147 months) for non-Hurler patients. For MPS III (*n* = 46), the median diagnostic delay was 33 months (range 1-365 months). No difference was observed between the RP and SP phenotypic groups. Comparing the diagnostic delay over time using 5-year time intervals, no reduction in the diagnostic delay was observed for MPS I or MPS III.

**Conclusions:**

In the Netherlands, the time to diagnosis for patients with MPS I and MPS III has not changed between 1988 and 2017, and an extensive delay still exists between the first visit to a medical doctor for disease-related symptoms and the final diagnosis. The numerous campaigns launched to increase awareness, leading to earlier diagnosis of these rare disorders, particularly of MPS I, have failed to achieve their goal. Robust selected screening protocols embedded in national guidelines and newborn screening for disorders that meet the criteria for population screening may be the only effective approaches for reducing the diagnostic delay.

## Background

Rare diseases with a prevalence of less than 1 in 2000 citizens (as defined by the European Commission; EC) often carry a high physical and psychological burden and impact the quality of life of the patients, parents and caregivers. More than 6000 rare diseases have been identified, and >50% are present during childhood (https://ec.europa.eu/health/rare_diseases/policy, http://www.eurordis.org/sites/default/files/publications/Fact_Sheet_RD.pdf). During previous decades, public and non-public organizations have launched numerous initiatives to increase the awareness of rare diseases, and in 1999, rare diseases first appeared on the agenda of the EC, resulting in a set of regulations and policies focusing on improving the recognition and visibility of rare diseases (https://ec.europa.eu/health/rare_diseases/policy, http://ec.europa.eu/health/archive/ph_overview/previous_programme/rare_diseases/raredis_wpgm99_en.pdf).

Due to their nature and the non-specific symptoms at presentation and during the early phases of the disease, rare diseases are often un- or misdiagnosed for extended periods, leading to a long diagnostic delay [1–4]. Patients may visit many different healthcare professionals and undergo multiple unnecessary investigations before the correct diagnosis is finally achieved [1–4]. This diagnostic odyssey may significantly add to the burden of the disease [1, 2, 4]. An early diagnosis is particularly essential if a disease-modifying treatment is available because the patients’ outcome often depends on the timely initiation of treatment [5–7]. Finally, because approximately 80% of rare diseases are inherited, an early diagnosis may allow genetic counseling and informed decision-making in family planning (https://www.eurordis.org/sites/default/files/publications/Fact_Sheet_RD.pdf).

To prevent unnecessarily delayed diagnoses, numerous campaigns have been launched to increase awareness of rare diseases. Many campaigns, such as the ‘rare diseases day’ initiative, which has become a yearly event in many countries worldwide, are of a general nature, raising awareness of the existence of ‘rare diseases’. Other initiatives focus on specific diseases and promoting an early diagnosis, thereby allowing the timely initiation of treatment [8–10] (http://www.rarediseaseday.org/events/world). These campaigns are organized by patient advocacy groups, health care providers and pharmaceutical companies.

However, to the best of our knowledge, no studies have specifically investigated whether these campaigns have reduced the diagnostic delay. We investigated the time to diagnosis of two very rare, invariable progressive and severe, inborn errors of metabolism: mucopolysaccharidosis type I (MPS I; estimated birth prevalence 1:100,000) for which treatment has been available for more than 15 years, and mucopolysaccharidosis type III (MPS III; estimated birth prevalence 1:60,000) for which treatment is under study (Table 1). Both disorders belong to the group of lysosomal storage disorders. We assessed whether the diagnostic delay has decreased over recent decades.

**Table 1:** Symptoms frequently observed in MPS I and MPS III patients and information regarding the different phenotypes and enzymatic subtypes

Disease	OMIM	Enzyme deficiency	Storage material	Main clinical features	Treatment	Prevalence
Mucopolysaccharidosis type 1 (MPS I)						
*MPS I – Hurler (MPS I-H)*	607,014	α-L-iduronidase (IDUA)	Dermatan sulfate (DS) and heparan sulfate (HS)	Progressive neurocognitive decline, hernias, facial dysmorphisms, corneal clouding, stiff joints, dysostosis multiplex, cardiac problems and hepatosplenomegaly. Death in childhood if untreated.	HSCT	1.07/1.19 per 100.000 newborns
*MPS I – Hurler-Scheie (MPS I-H/S)*	607,015			Phenotype intermediate between MPS I-H and MPS I-S. Can present with or without neuronopathic disease.	HSCT or ERT	
*MPS I – Scheie (MPS I-S)*	607,016			Corneal clouding, stiff joints, mild dysostosis multiplex. Normal intelligence en life expectancy.	ERT	
Mucopolysaccharidosis type 3 (MPS III)						
*MPS IIIA*	252,900	Heparan N-sulfatase (SGSH)	Heparan sulfate (HS)	Progressive neurocognitive decline, behavioral problems, sleep disturbances, progressive loss of motor functions. Death in second or third decade of life. Broad spectrum of disease severity.	Not available	1.52/1.89 per 100.000 newborns
*MPS IIIB*	252,920	*N*-acetyl-α-glucosaminidase (NAGLU)				
*MPS IIIC*	252,930	Acetyl CoA:α-glucosaminide *N*-acetyltransferase (HGSNAT)				
*MPS IIID*	252,940	*N*-acetylglucosamine 6-sulfatase (GNS)				

## Methods

### Patients

This single center study was conducted at the Academic Medical Center (AMC) in Amsterdam and involved interviews with patients and/or parents or legal guardian(s) of patients with MPS I and MPS III with a confirmed diagnosis since 1988. Before 1988, reliable data were unavailable. The data were verified and/or supplemented with chart reviews or data inquiries from the general practitioner (GP) and the medical specialist(s) visited prior to diagnosis. Our center is a center of expertise for MPS I and MPS III in the Netherlands.

All MPS I and MPS III patients were included regardless of the phenotype. Table 1 presents the symptoms frequently observed in MPS I and MPS III patients and information regarding the different phenotypes and enzymatic subtypes [11–15]. The phenotypes were assessed by an experienced clinician (FAW) based on the available clinical data. Only patients with a diagnosis confirmed by enzymatic testing and/or a mutation analysis were included. Patients were only included if the diagnostic studies leading to the final diagnosis were based on the clinical symptoms. Patients who underwent diagnostic studies because of an affected family member were excluded. All patients and/or their parents or legal guardians provided informed consent for this study. The study proposal was reviewed by the Medical Ethics Committee of the AMC, who deemed that formal ethical approval was not necessary for this study.

### Data collection

The data were collected using structured telephone interviews with patients and/or the patients’ parents or legal guardian(s). The following variables were recorded:

- Year/month of first visit to the GP for a symptom that was, in hindsight, likely related to MPS I/MPS III.

- Year/month of first referral visit to a medical specialist for a symptom that was, in hindsight, likely related to MPS I/MPS III.

- Year/month of the confirmatory diagnosis, which was defined by the first demonstration of deficient enzyme activity or the presence of disease causing mutations.

From each of these visits, the following data were recorded:

- MPS I/MPS III-related symptom leading to the visit.

- Other MPS I/MPS III-related symptoms present at that time point.

- Type of medical specialist visited at first referral for a disease-related symptom.

- Type of medical specialist who made the diagnosis.

MPS I and MPS III disease-related symptoms are presented in Table 2.

**Table 2:** Disease-related symptoms for MPS I and MPS III

Disease-related symptoms	
Mucopolysaccharidosis type I	Mucopolysaccharidosis type III
*Hernias* • Inguinal hernia • Umbilical hernia	*Developmental delay or decline* • Neurocognitive functions • Motor functions
*Ear, nose, throat problems* • Frequent upper airway infections • Obstructive sleep apneas or excessive snoring during sleep • Tympanostomy tubes • Adenoidectomy • Tonsillectomy	*Behavioral problems* • Hyperactivity/restlessness • Aggression • Anxiety • Autistic behaviors • Other
*Gastro-intestinal problems* • Hepatosplenomegaly	*Dysmorphic features* • Coarse facial features • Coarse hair • Hirsutism • Other
*Cardiac problems* • Cardiomyopathy • Valvular dysfunction	
*Skeletal and joint problems* • Joint stiffness • Skeletal deformities • Kyphosis • Hip dysplasia • Bullet shaped metacarpals • Stunted growth of the long bones • Broad oar shaped ribs • Short stature • Carpal tunnel syndrome • Trigger fingers • Tendon shortening • Early arthrosis	*Ear, nose, throat problems* • Frequent upper airway infections • Frequent ear infections • Hearing problems • Tympanostomy tubes • Adenoidectomy • Tonsillectomy
	*Gastro-intestinal problems* • Frequent diarrhea • Hepatomegaly • Other
*Hydrocephalus*	*Sleeping problems*
*Corneal clouding*	*Seizures*
*Dysmorphic features* • Frontal bossing • Depressed nasal bridge • Full lips • Macroglossia	*Hernias* • Inguinal hernia • Umbilical hernia
*Developmental delay*	

### Statistical analyses

The statistical analyses were performed using SPSS software for Windows (version 23.0, SPSS Inc., Chicago, Illinois, USA). Non-parametric Mann-Whitney U tests were performed to assess the significant differences in the time between the first visit to the GP and diagnosis and the time between the first visit to a medical specialist and the final diagnosis within the cohort of MPS I patients and between the Hurler and non-Hurler patients. The same analyses were performed for the RP and SP MPS III patients.

To assess whether the diagnostic delay changed over time, the MPS I and MPS III patients were divided into different groups based on the year of diagnosis using a 5-year time interval. Non-parametric Kruskall-Wallis tests were performed to assess the significant differences among these groups.

## Results

### MPS I and MPS III patient characteristics

Thirty-two MPS I patients met the inclusion criteria; of these patients, three were excluded (two patients did not consent, and one was lost to follow-up). From the group of MPS III patients, 53 patients met the inclusion criteria, and 7 of these patients were lost to follow-up. The characteristics of the patients included in the study are provided in Table 3. At the time of this study, one male MPS IIIA patient (aged 4 years and 9 months) was considered too young to reliably predict the phenotypic severity.

**Table 3:** Characteristics of the MPS I and MPS III patients. At the time of this study, one of the MPS III patients (aged 4 years and 9 months) was considered too young to determine the phenotypic severity

Patient characteristics			
MPS I	N	MPS III	N
Total number of patients	29	Total number of patients	46
Male	15	Male	27
Female	14	Female	19
MPS I phenotype		MPS III subtype	
Hurler	20	MPS IIIA	28
Non-Hurler	9	MPS IIIB	9
		MPS IIIC	9
		MPS III phenotype	
		Rapidly progressing (RP) MPS III	16
		Slowly progressing (SP) MPS III	28
		Unknown	1

### MPS I: First visit to the GP for an MPS I-related symptom

Sixteen of the 29 MPS I patients first visited a GP for an MPS I-related symptom and were subsequently referred to a medical specialist. Eleven patients were directly seen by a medical specialist for MPS I-related symptoms without a prior visit to the GP, and this information was unclear for 2 patients. Due to the small group size, no further analyses of the first visit to the GP were performed.

### MPS I: First visit to a medical specialist for an MPS I-related symptom

The MPS I patients first visited a medical specialist for an MPS I-related symptom at a median age of 4 months (range 0 – 54 months; median age: MPS I Hurler patients 3 months (range 0 – 20 months) and MPS I non-Hurler patients 12 months (range 0 – 54 months)) (Table 4).

**Table 4:** Characteristics of the entire group of MPS I patients at the first visit to a medical specialist for an MPS I-related symptom as specified for the MPS I Hurler and non-Hurler patients

First visit to a medical specialist						
	All MPS I		Hurler		Non-Hurler	
Number of patients	29		20		9	
Age at first visit (months)						
Median	4		3		12	
Range	0 – 54		0 – 20		0 – 54	
Specialism of 1st referral	Nr.	%	Nr.	%	Nr.	%
Ear, nose, and throat specialist	3	10%	2	10%	1	11%
General pediatrician	20	69%	14	70%	6	67%
Orthopedic surgeon	2	7%	1	5%	1	11%
Pediatric surgeon	3	10%	2	10%	1	11%
Pediatric cardiologist	1	3%	1	5%	0	0%
Symptom leading to 1st referral	Nr.	%	Nr.	%	Nr.	%
Recurrent airway infections	7	24%	5	25%	2	22%
Upper airway obstruction	3	10%	2	10%	1	11%
Inguinal/umbilical hernia	4	14%	3	15%	1	11%
Hydrocephalus	2	7%	1	5%	1	11%
Hepatosplenomegaly	1	3%	1	5%	0	0%
Kyphosis/hip dysplasia	3	10%	2	10%	1	11%
Joint stiffness	1	3%	0	0%	1	11%
Facial features	4	14%	4	20%	0	0%
Hearing problems	1	3%	1	5%	0	0%
Growth delay	2	7%	1	5%	1	11%
Developmental delay	1	3%	0	0%	1	11%
Other MPS I-related symptoms at 1st referral	Nr.	%	Nr.	%	Nr.	%
Recurrent airway infections	7	24%	4	20%	3	33%
Upper airway obstruction	10	34%	9	45%	1	5%
Inguinal/umbilical hernia	8	28%	6	30%	2	10%
Hydrocephalus	2	7%	2	10%	0	0%
Hepatosplenomegaly	2	7%	1	5%	1	5%
Joint stiffness	3	10%	2	10%	1	11%
Facial features	4	14%	2	10%	2	10%
Hearing problems	6	21%	4	20%	2	10%
Vision problems	1	3%	0	0%	1	5%
Developmental delay	5	5%	2	10%	3	15%
Growth delay	1	3%	0	0%	1	5%

The sums of the percentages of each item may not equal 100% because the percentages represent rounded values

Both the Hurler and non-Hurler patients were first seen by a general pediatrician (69%), and recurrent airway infections were the most common reason for these visits. Additional MPS I-related symptoms that were present at the time of the first visit to a medical specialist are presented in Table 4.

### MPS I: Time to diagnosis

**Fig. 1:**
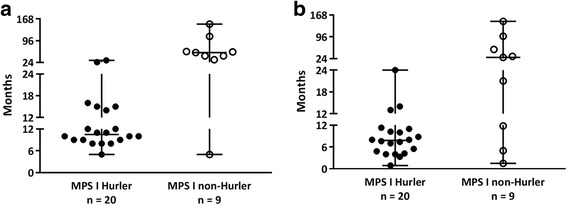
**a** Age at diagnosis of the MPS I Hurler and non-Hurler patients. **b** Time between the first visit to a medical specialist for an MPS I-related symptom and final diagnosis in MPS I Hurler and non-Hurler patients. In all figures, time is presented in months

The median age at diagnosis of all MPS I patients was 12 months (range 5 – 151 months) (Table 5). The Hurler patients were diagnosed at a significantly younger age (11 months (range 5 – 31 months) than the non-Hurler patients (57 months (range 5 – 151 months) (*p* = 0.005) (Fig. 1a). The diagnosis of MPS I was most often made by a general pediatrician (45%), followed by a pediatrician specialized in inborn errors of metabolism (IEM) (31%).

**Table 5:** Characteristics of the entire group of MPS I patients, MPS I Hurler patients and non-Hurler patients at the time of diagnosis

Final diagnosis of MPS I						
	All MPS I		Hurler		Non-Hurler	
Number of patients	29		20		9	
Age at diagnosis (months)						
Median	12		11		57	
Range	5 – 151		5 – 31		5 – 151	
Delay medical specialist - diagnosis (months)						
Median	9		8		28	
Range	1 – 147		1 – 24		2 – 147	
Diagnosing specialist	Nr.	%	Nr.	%	Nr.	%
General pediatrician	13	45%	10	50%	3	33%
Clinical geneticist	4	14%	3	15%	1	11%
Pediatrician specialized in IEM	9	31%	5	25%	4	44%
Ophthalmologist	2	7%	2	10%	0	0%
Rheumatologist	1	3%	0	0%	1	11%

The sums of the percentages of each item may not equal 100% because the percentages represent rounded values. IEM: inborn errors of metabolism

The median delay between the first visit to a medical specialist and the final diagnosis for the entire group was 9 months (range 1 – 147; median delay: Hurler patients 8 months (range 1 – 24 months) and non-Hurler patients 28 months (range 2 – 147 months; the difference between the Hurler and non-Hurler patients was not significant) (Fig. 1b).

**Fig. 2:**
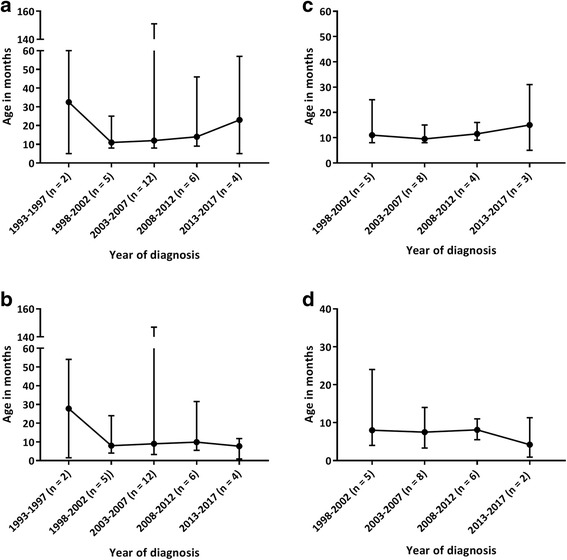
**a** Age at diagnosis of the entire group of MPS I patients. **b** Time between the first visit to a medical specialist for an MPS I-related symptom and final diagnosis in the entire group of MPS I patients. **c** Age at diagnosis of the group of MPS I Hurler patients. **d** Time between the first visit to a medical specialist for an MPS I-related symptom and final diagnosis in the group of MPS I Hurler patients. In all figures, time is presented in months. Both MPS I and MPS I Hurler patients were divided into groups based on the year of diagnosis

To assess whether the diagnostic delay changed over time, the patients were divided into different subgroups based on the year of the diagnosis using a 5-year time interval. Over the study period from 1988 to 2017, no significant reduction in the diagnostic delay was observed (Fig. 2a). In addition, no significant differences were observed in the time between the first visit to the medical specialist and the final diagnosis (Fig. 2b). When performing the same analyses only for the group of Hurler patients, no differences were observed in the median age at diagnosis and the median time between the first visit to the medical specialist and diagnosis over time (Fig. 2b and d).

### MPS III: First visit to the GP for an MPS III-related symptom

Almost all MPS III patients (45 of the 46) first visited a GP for an MPS III-related symptom (Table 6). The median age at the first visit for the entire group was 22 months (range 1 – 84 months): 16 months for the RP patients (range 1 – 33 months) and 24 months for the SP patients (range 1 – 84 months). Upper airway infections and middle ear problems were the most frequent symptoms leading to the visit to the GP. Other symptoms leading to the visit to the GP and additional MPS III-related symptoms present at that time are presented in Table 6.

**Table 6:** Characteristics of the entire group of MPS III patients, RP MPS III patients and SP MPS III patients at the first visit to the GP for an MPS III-related symptom

First visit to general practitioner						
	All MPS III		RP MPS III		SP MPS III	
Number of patients	45		16		28	
Age at 1st visit (months)						
Median	22		16		24	
Range	1 – 84		1 – 33		1 – 84	
Symptom leading to 1st visit	Nr.	%	Nr.	%	Nr.	%
Developmental delay	9	20%	2	13%	6	21%
Upper airway problems	30	67%	12	75%	18	64%
Diarrhea	1	2%	0	0%	1	4%
Liver problems	1	2%	0	0%	1	4%
Seizures	1	2%	0	0%	1	4%
Inguinal/umbilical hernia	3	7%	2	13%	1	4%
Other MPS III-related symptoms at 1st presentation	Nr.	%	Nr.	%	Nr.	%
Developmental delay	15	33%	3	19%	12	43%
Behavioral problems	29	64%	10	63%	18	64%
Dysmorphic features	27	60%	11	69%	15	54%
Upper airway problems	10	22%	2	13%	7	25%
Diarrhea	23	51%	11	69%	11	39%
Sleeping disturbances	21	47%	9	56%	11	39%
Inguinal/umbilical hernia	11	24%	8	50%	3	11%

One of the SP MPS III patients did not visit the GP before receiving a referral to a medical specialist. One of the patients was considered too young to determine the phenotypic severity at the time of this study. The sums of the percentages of each item may not equal 100% because the percentages represent rounded values

### MPS III: First visit to a medical specialist for an MPS III-related symptom

The median age at the first visit to a medical specialist for an MPS III-related symptom was 28 months in the MPS III patients (range 2 – 171 months; median age: 19 months in RP patients (range 6 – 39 months) and 30 months in SP patients (range 2 – 171 months)) (Table 7). The patients were most often referred to an ear, nose, and throat (ENT) specialist (65%), and 61% of the cases subsequently underwent an adenotonsillectomy or placement of tympanostomy tubes (81% of the RP patients and 52% of SP patients). In most patients, other MPS III-related symptoms were already present at the time of the first visit to the medical specialist, including developmental delay, behavioral and sleeping problems, dysmorphic features, hernias and recurrent episodes of unexplained diarrhea.

**Table 7:** Characteristics of the entire group of MPS III patients, RP MPS III patients and SP MPS III patients at the first visit to a medical specialist for an MPS III-related symptom

First visit to a medical specialist						
	All MPS III		RP MPS III		SP MPS III	
Number of patients	46		16		29	
Age at 1st visit (months)						
Median	28		19		30	
Range	2 – 171		6 – 39		2 – 171	
Specialism of 1st referral	Nr.	%	Nr.	%	Nr.	%
Ear, nose, and throat specialist	30	65%	13	81%	16	55%
General pediatrician	7	15%	1	6%	6	21%
Pediatric neurologist	2	4%	0	0%	2	7%
Pediatric surgeon	4	9%	2	13%	2	7%
Pediatric cardiologist	1	2%	0	0%	1	3%
Pediatric psychiatrist	2	4%	0	0%	2	7%
Symptom leading to 1st referral	Nr.	%	Nr.	%	Nr.	%
Developmental delay	7	15%	0	0%	7	24%
Upper airway problems	2	4%	1	6%	1	3%
Adenotonsillectomy/tympanostomy tubes	29	63%	13	81%	15	52%
Diarrhea	1	2%	0	0%	1	3%
Liver problems	1	2%	0	0%	1	3%
Seizures	1	2%	0	0%	1	3%
Correction Inguinal/umbilical hernia	4	9%	2	13%	2	7%
Cardiac murmur	1	2%	0	0%	1	3%
Other MPS III-related symptoms at 1st referral	Nr.	%	Nr.	%	Nr.	%
Developmental delay	21	46%	10	63%	10	34%
Behavioral problems	35	76%	14	88%	20	69%
Dysmorphic features	29	63%	11	69%	17	59%
Upper airway problems	10	22%	1	6%	9	31%
Diarrhea	23	50%	11	69%	11	38%
Sleeping disturbances	21	46%	9	56%	12	41%
Inguinal/umbilical hernia	11	24%	9	56%	2	7%

The sums of the percentages of each item may not equal 100% because the percentages represent rounded values

### MPS III: Time to diagnosis

**Fig. 3.**
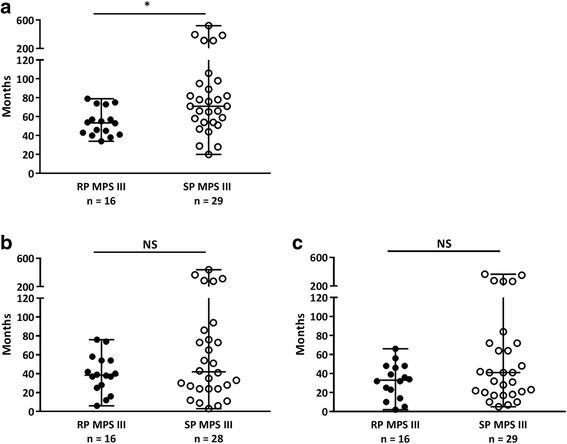
**a** Age at diagnosis in the RP and SP MPS III patients. **b** Time between the first visit to the GP for an MPS III-related symptom and the final diagnosis (in months) in the RP and SP MPS III patients. **c** Time between the first visit to a medical specialist for an MPS III-related symptom and the final diagnosis (in months) in the RP and SP MPS III patients. * *p* < 0.05; NS = non-significant

The final diagnosis was established at a median age of 62 months, with a range of 20 to 522 months (Table 8). As shown in Fig. 3a, the RP patients were significantly younger at the time of diagnosis (54 months, range 34 – 79 months) than the SP patients (71 months, range 20 – 522) (*p* < 0.05). The patients were most often diagnosed by a clinical geneticist, followed by a general pediatrician or a pediatrician specializing in IEM.

**Table 8:** Characteristics of the entire group of MPS III patients, RP MPS III patients and SP MPS III patients at the time of diagnosis

Final diagnosis of MPS III						
	All MPS III		RP MPS III		SP MPS III	
Number of patients	46		16		29	
Age at diagnosis (months)						
Median	62		54		71	
Range	20 – 522		34 – 79		20 – 522	
Delay general practitioner – diagnosis (months)						
Median	39		39		42	
Range	2 – 438		6 – 76		3 – 438	
Delay medical specialist – diagnosis (months)						
Median	33		33		41	
Range	1 – 365		2 – 66		5 – 365	
Diagnosing specialist	Nr.	%	Nr.	%	Nr.	%
Clinical geneticist	16	35%	5	31%	11	38%
General pediatrician	13	28%	5	31%	8	28%
Pediatrician specialized in IEM	12	26%	5	31%	6	21%
Pediatric neurologist	4	9%	1	6%	3	10%
Specialist for the mentally disabled	1	2%	0	0%	1	3%

The sums of the percentages of each item may not equal 100% because the percentages represent rounded values. IEM: inborn errors of metabolism

The median delay between the first visit to the GP for an MPS III-related symptom and the final diagnosis in the entire group of MPS III patients was 39 months (range 2 – 438 months), and no difference was observed among patient groups with varying disease severities (Fig. 3b). The median time between the first visit to a medical specialist for an MPS III-related symptom and the final diagnosis was 33 months (range 1 – 365 months). Similarly, no difference in delay was observed between the two phenotypic groups (Fig. 3c).

**Fig. 4:**
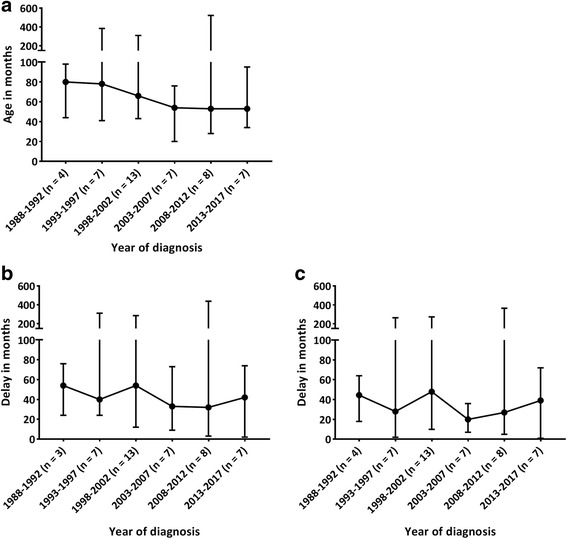
**a** Age at diagnosis in the MPS III patients. **b** Time between the first visit to a GP for an MPS III-related symptom and the final diagnosis. **c** Time between the first visit to a medical specialist for an MPS III-related symptom and the final diagnosis. In all figures, time is presented in months. The MPS III patients were divided into groups based on the year of diagnosis. One patient never visited the GP for an MPS III-related symptom

To assess whether the diagnostic delay changed over time, the MPS III patients were divided into different groups based on the year of the diagnosis using a 5-year time interval. Although a trend of diagnosing at a younger age was observed over time (Fig. 4a), no significant differences were observed between the cohorts of patients diagnosed in different time intervals. Similarly, the time between the first visit to the GP for an MPS III-related symptom and the time of the final diagnosis (Fig. 4b) and the time between the first visit to a medical specialist and the time of the final diagnosis (Fig. 4c) were not significantly reduced during the study period from 1988 to 2017. Further analyses of the RP and SP patients did not reveal any differences over time (data not shown).

## Discussion

This study is the first to report the diagnostic odyssey in MPS I and MPS III patients in the Netherlands. We demonstrate the presence of a substantial diagnostic delay in both MPS I and MPS III patients without a reduction in the time between the first consultation with a medical doctor (GP or medical specialist) for disease-related symptoms and the time of the final diagnosis over a 20-year period.

In the Dutch healthcare system, patients, including children, are typically first seen by a GP, who may refer the patient to a medical specialist. Thus, the time to diagnosis after the visit to the GP was longer than the time between the visit to a medical specialist and the diagnosis. Remarkably, the longest diagnostic delay was observed after the first visit to a medical specialist, particularly in the MPS III patients.

The MPS I patients were diagnosed at a significantly younger age than the MPS III patients, which is most likely due to the early manifestation of the somatic symptoms [11, 16], leading to earlier medical attention and referral. MPS I patients with the severe Hurler phenotype were diagnosed at a significantly younger age than the non-Hurler patients. The median age at diagnosis in the Hurler patients was comparable to that reported in previous studies [2, 17–19]. However, the more attenuated non-Hurler patients in our cohort were diagnosed at an earlier age than that reported in other studies [14, 17–20]. This finding may be due to the relatively small sample size of non-Hurler patients in our cohort. The lack of a decrease in the time to diagnosis over the previous two decades is disappointing and worrisome for two reasons. First, an early diagnosis allows for the early initiation of treatment and better disease outcomes. Treatment with hematopoietic cell transplantation (HCT) for MPS I Hurler was first shown to be effective in halting or preventing the cognitive decline in the early 1980s and is currently the treatment of choice for this group of patients. Earlier HCT leads to better outcomes [5, 21, 22]. In addition, intravenous enzyme replacement therapy (ERT) is the treatment of choice for MPS I patients with a non-Hurler phenotype, and studies have demonstrated that an early start of treatment is beneficial [6, 7, 23, 24]. Second, to reduce the diagnostic delay and promote early diagnosis, numerous MPS I awareness campaigns have been launched, particularly after the introduction of ERT for the treatment of the somatic symptoms in 2003. These campaigns included direct mailings to health care professionals in the Netherlands presenting the typical features of MPS I patients, expert lectures on early symptoms of MPS I at scientific meetings of relevant medical specialists (including pediatricians, ENT specialists, pediatric rheumatologists and pediatric neurologists) and exhibit booths of a pharmaceutical company commercially marketing ERT for MPS I (Genzyme Sanofi) providing educational material on lysosomal storage disorders, including MPS I, at major relevant medical conferences in the Netherlands. Our data indicate that these efforts have not led to a significant reduction in the time to an MPS I diagnosis.

In our cohort of MPS III patients, the diagnosis was established at a significantly younger age in the severe RP patients (age 54 months; 4 years and 6 months) than in the SP patients (age 71 months; 5 years and 11 months). However, the diagnostic process preceding the diagnosis did not differ between the two groups, and the age at final diagnosis is comparable to observations reported in other studies [25–27]. Although no disease-modifying treatment is currently available, several clinical trials, including intrathecal ERT and gene therapy, have recently been initiated for MPS III types A and B [28, 29]. An early diagnosis and early start of treatment before the onset of progressive cognitive deterioration are considered essential. Given that patients with the RP phenotype plateau in development by 30 months and exhibit rapid cognitive decline at 40 – 50 months, a diagnosis should be made before the age of 3 years to allow the initiation of therapy at the optimal timing [13]. This goal, however, was only achieved in 9% of the patients in this study, and no decrease in age at diagnosis was observed over the previous 20 years.

Our study has some limitations. First, we defined diagnostic delay as the time between the first visit to a GP or medical specialist for a potential disease-related symptom and the final diagnosis, whereas diagnostic delay generally refers to the time between the onset of symptoms and diagnosis in other studies [14, 30, 31]. However, we consider the use of the time of symptom onset susceptible to a significant recall bias, whereas the time of the first visit to a medical doctor can be verified, thereby providing more reliable data. Second, our study has a retrospective design. Nevertheless, the amount of missing data was small, and the data could be verified in the medical records. In addition, due to the rarity of both disorders, a prospective design is not feasible. Third, the number of patients included in our study was small. Given that we were able to recruit almost all patients from the Netherlands diagnosed with MPS I and MPS III between 1988 and 2017, we assume that our data reliably represent the situation in our country. Larger scale, multi-national, studies on the diagnostic delay in patients with MPS or other rare or ultra-rare diseases are needed to corroborate our findings. In Europe, such studies may be initiated by the recently established European Reference Networks for rare diseases (ERNs) (https://ec.europa.eu/health/ern_en). Finally, MPS I and MPS III are ultra-rare (ultra-orphan) diseases because they affect less than one person per 50,000 people (http://eur-lex.europa.eu/legal-content/EN/TXT/?uri=CELEX:32014R0536). The results of our study might not be applicable to relatively more common rare diseases affecting one person per 2000 – 50,000 people.

The lack of a reduction in the diagnostic delay over time was previously reported for MPS I by d’Aco et al.*,* based on data from an observational international MPS I registry [18]. In addition, a study investigating the time of diagnosis in Pompe disease, which is a lysosomal storage disease in which the timing of the start of therapy (ERT) is essential, to the surprise of the authors, also failed to demonstrate a reduction in the diagnostic delay despite improved diagnostic laboratory techniques allowing for a rapid diagnosis [32]. Multiple efforts to increase awareness of Pompe disease and expedite its diagnosis have been exerted globally over recent decades.

Determining why awareness campaigns for rare diseases fail to reduce the diagnostic delay in MPS I and III and Pompe disease is challenging. Due to the very low birth prevalence of these disorders, many specialists, including GPs, general pediatricians, orthopedic surgeons and ENT specialists, may visit with no or only one undiagnosed patient during their entire career. Awareness of specific (combinations of) symptoms of a (ultra) rare disease may be lacking when confronted with a patient (many) years after exposure to an awareness campaign. Long-lasting knowledge regarding the symptoms of (ultra) rare diseases can likely only be achieved by intensive repetitive learning, which is not a feasible option for all medical specialists. Furthermore, because most symptoms at presentation are not specific, considerable time is generally spent excluding more common disorders.

Several alternative strategies are possible. One strategy involves the selective screening of groups of patients with certain symptoms but without a diagnosis of the rare disease of interest. Such studies have been performed for MPS I and included studies investigating MPS screening in patients with previous surgical repair or the presence of inguinal and/or umbilical hernia in combination with pediatric ENT surgery and children visiting rheumatology, hand or skeletal dysplasia clinics (clinicaltrials.gov identifiers: NCT02095015, NCT01675674). Both trials have been terminated. To the best of our knowledge, these results have not been published, suggesting a failure to identify significant numbers of otherwise unrecognized patients. A study investigating screening patients under the age of 18 years with carpal tunnel syndrome for MPS also failed to detect patients with MPS [33]. The extremely low yield of screening certain groups of patients for an ultra-rare disorder likely discourages participation, leading to the discontinuation of these programs. The yields of selective screening may improve when groups of patients are screened for a multitude of disorders, thus obviating the need of knowledge regarding specific rare disorders. Because the diagnostic approach in children with impaired cognitive development may significantly differ among health care systems in different regions of the world and obtaining an early diagnosis in patients with MPS III is very difficult, screening of children with an intellectual developmental disorder for several rare diseases may significantly reduce the diagnostic delay. A diagnostic algorithm for the identification of treatable causes of cognitive impairment has been proposed [34], and several publications have demonstrated the importance of an early metabolic screening in all patients with unexplained developmental delay [35, 36]. In addition, a review by Cleary and Green [37] provided a guideline for the metabolic screening of patients with a developmental delay. The authors emphasize that IEMs can present with isolated developmental delay and that any regression of skills is suggestive of an IEM and warrants an intensive metabolic investigation. The slowing of cognitive development with a speech delay is one of the first symptoms of MPS III and often occurs before the age of 2.5 years; these symptoms could lead to an early diagnosis if these guidelines are followed. However, as the median age at diagnosis of patients with the most common RP phenotype is 54 months (range 34 – 79 months) in our study, it is clear that these guidelines are not used in the Netherlands. Indeed, the current guideline by the Dutch Society for Pediatrics (NvK, 2005) recommends screening for IEMs only if additional symptoms are present and not in in the presence of isolated cognitive delay (https://www.nvk.nl/Portals/0/richtlijnen/mentale%20retardatie/mentaleretardatie.pdf). Fortunately, a new guideline is currently under development.

An interesting option for the (near) future is computer-assisted diagnosis, which can expedite the diagnosis of rare diseases. Artificial intelligence, deep learning and even a 3D facial analysis may assist clinicians during the diagnostic process, suggesting both diagnoses and appropriate investigations based on information in the electronic patient records [38–40].

Finally, newborn population screening (NBS) may ensure very early diagnosis in patients with rare diseases and should be considered if a disease meets at least the following criteria (first proposed by Wilson and Jungner in 1968) [41]: (a) the condition is an important health problem; (b) a suitable test for diagnosis is available; (c) a latent or early symptomatic state is recognizable; (d) the understanding of the condition’s natural history is adequate; and (e) an acceptable treatment for patients with a recognized disease is available. Because MPS I is considered to meet these criteria, this disorder has been introduced in NBS programs in the USA and Taiwan [42] and will be introduced in the NBS panel in the Netherlands (https://zoek.officielebekendmakingen.nl/blg-775624.pdf). However, this will lead to new challenges, including the detection of pseudo deficiencies for MPS I, as well the challenges often associated with newborn screening such as uncertain diagnoses and the inability to predict the phenotype, which may lead to significant emotional burden [43–45]. MPS III is currently not considered eligible for NBS because no disease-modifying therapy is yet available.

## Conclusions

In conclusion, we demonstrate that the time to diagnosis in patients with MPS I and MPS III has not changed between 1988 and 2017 in the Netherlands and a long delay between the first visit to a medical doctor for symptoms related to the disease and the final diagnosis is common. Therefore, campaigns to increase the awareness of rare diseases in general, and of MPS I specifically, failed to achieve this goal. This finding is likely due to the non-specific initial symptoms and the ultra-rare nature of both disorders. Because most medical doctors will probably visit with patients with these disorders never or only once during their entire career, it is questionable whether education of combinations of symptoms of specific (ultra) rare diseases will ever be effective. Robust selected screening protocols embedded in national guidelines may be the best alternative. Such guidelines may include urinary screening for glycosaminoglycans in all children with kyphosis and extensive screening for IEMs in all children with developmental delay, thus obviating the need for detailed knowledge regarding specific (ultra) rare diseases. Finally, NBS should be considered for those disorders that meet the criteria for population screening because this may be the only approach to guarantee a timely initiation of therapy in all patients with specific rare diseases.

## Abbreviations

AMC: Academic Medical Centre
EC: European Commission
ENT: Ear, nose, and throat
ERT: Enzyme replacement therapy
GP: General practitioner
HCT: Hematopoietic cell transplantation
IEM: Inborn errors of metabolism
MPS: Mucopolysaccharidosis
NBS: Newborn screening
NS: Non-significant
RP: Rapidly progressing
SP: Slowly progressing
USA: United States of America
